# Evaluation of Lercanidipine in Paclitaxel-Induced Neuropathic Pain Model in Rat: A Preliminary Study

**DOI:** 10.1155/2012/143579

**Published:** 2012-03-25

**Authors:** Lekha Saha, Debasish Hota, Amitava Chakrabarti

**Affiliations:** Department of Pharmacology, PGIMER, Chandigarh 160012, India

## Abstract

*Objective*. To demonstrate the antinociceptive effect of lercanidipine in paclitaxel-induced neuropathy model in rat. *Materials and Methods*. A total of 30 rats were divided into five groups of six rats in each group as follows: Gr I: 0.9% NaCl, Gr II: paclitaxel + 0.9% NaCl, Gr III: paclitaxel + lercanidipine 0.5 *μ*g/kg, Gr IV: paclitaxel + lercanidipine 1 *μ*g/kg, and Gr V: paclitaxel + lercanidipine 2.5 *μ*g/kg. Paclitaxel-induced neuropathic pain in rat was produced by single intraperitoneal (i.p.) injection of 1 mg/kg of paclitaxel on four alternate days (0, 2, 4, and 6). The tail flick and cold allodynia methods were used for assessing the pain threshold, and the assessments were done on days 0 (before first dose of paclitaxel) and on days 7, 14, 21, and 28. *Results*. There was a significant decrease (*P* < 0.001) in the tail flick and cold allodynia latency in the paclitaxel-alone group from day 14 onward when compared with day 0. In the lercanidipine groups, the decrease in the tail flick and cold allodynia latency was not observed in 1.0 and 2.5 *μ*g/kg groups and it was statistically significant (*P* < 0.01) when compared with paclitaxel-alone group.

## 1. Introduction

Neuropathic pain is a type of pain which is caused by damage to or dysfunction of the nervous system. [1] Neuropathic pain cannot be explained by a single disease process or a single specific location of damage. It is a disorder in the structure and function of peripheral, motor, sensory, and/or autonomic neurons either partially or completely [2]. Neuropathic pain may be divided into peripheral, central, or mixed (peripheral and central) neuropathic pain. As much as 7% to 8% of the population is affected and in 5%, it may be severe [3].

*In order to evaluate the mechanisms of neuropathic pain and to find new therapeutic approaches, different experimental neuropathic pain models have been developed which include chronic constriction injury of sciatic nerve, partial sciatic nerve ligation, and partial transaction of sciatic nerve. *Current knowledge regarding the mechanisms of neuropathic pain is incomplete and is biased by a focus on animal models of peripheral nerve injury, and the treatment is often unsatisfactory. It is an area of largely unmet therapeutic need. The current pharmacological mainstays of clinical management are tricyclic antidepressants and certain anticonvulsants, but these only achieve clinically significant (>50%) pain relief in 40–60% of patients and are associated with several side effects. Opioids are generally considered to be less effective in neuropathic pain than in inflammatory pain [4].

*Paclitaxel, a commonly used antineoplastic drug can produce a dose-limiting painful peripheral neuropathy when given intraperitoneally and can be used as a model of peripheral neuropathy to evaluate the effect of various drugs in neuropathic pain [5].*

*Lercanidipine *is a third-generation L-type calcium channel blocker (CCB) belonging to the class dihydropyridines and is approved for the treatment of cardiovascular disorders. Various preclinical studies point to an altered role of voltage-gated Ca^2+^ channels, particularly the *α*-2 delta subunit after neuropathy, and specific blockers have been shown to differentially attenuate the behavioural hypersensitivities and altered dorsal horn neuronal responses that accompany experimental neuropathic pain. The studies done to evaluate the action of CCBs on CNS are quite limited [6].

In the published literatures, there is no data available regarding the modulation of paclitaxel-induced neuropathic pain by lercanidipine. Hence, the present study is undertaken to validate the model of neuropathic pain induced by paclitaxel and the effect of lercanidipine in this model of neuropathic pain.

## 2. Materials and Methods

### 2.1. Animals

Animals (Wistar rats weighing between 150–180 gm) were procured from the central animal house of Postgraduate Institute of Medial Education and Research (PGIMER), Chandigarh. They were kept individually in polypropylene cages in an environmentally controlled room of the departmental animal house. They were maintained at 25 ± 2°C with a 12-hour dark/light cycle and 40–70% humidity. The animals had free access to food and water and were fed with standard rat chow diet. Experiments were carried out after a week of acclimatization according to the guidelines of Animal Ethics Committee of the Institute and the Committee for the Purpose of Control and Supervision of Experimentation in Animals (CPCSEA) guidelines for animal care. Approval from the Institute's Animal Ethics Committee was taken prior to conducting the studies. Extreme care was taken to minimize the pain and discomfort to the animals.

### 2.2. Drugs and Chemicals

Paclitaxel (Sigma Chemical Company, St. Louis, USA) and lercanidipine (Ranbaxy, Delhi, India) were purchased for the study in the pure powder form. All the chemicals to be used in the experiment were of AR grade from reputed suppliers. All the drugs and chemicals were used as freshly prepared solutions and suspension. The solutions of paclitaxel were made in Dimethyl Sulfoxide (DMSO), and the solutions of lercanidipine were made in methanol.

### 2.3. Experimental Groups

A total of 30 rats were divided into five groups of six rats in each group as follows: Gr I: 0.9% NaCl, Gr II: paclitaxel +0.9% NaCl, Gr III: paclitaxel + lercanidipine 0.5 *μ*g/kg, and Gr IV: paclitaxel + lercanidipine 1 *μ*g/kg, Gr V: paclitaxel + lercanidipine 2.5 *μ*g/kg.

### 2.4. Paclitaxel-Induced Neuropathic Pain in Rat [5]

In this model, young Wistar rats were administered single intraperitoneal (i.p.) injection of 1 mg/kg of paclitaxel on four alternate days (0, 2, 4, and 6). The volume of injection was kept constant at 1 mL/kg. This model typically presents sensory neuropathy. The tail flick and cold allodynia methods were used for assessing the pain threshold, and the assessments were done on days 0 (before first dose of paclitaxel) and on days 7, 14, 21, and 28.

### 2.5. Treatment Protocol

As control, group I animals received only the normal diet with 0.9% NaCl alone and group II animals received Paclitaxel and 0.9% NaCl. From groups III to V, animals were administered single i.p. injection of Lercanidipine at doses of 0.5 *μ*g/kg, 1 *μ*g/kg and 2.5 *μ*g/kg, respectively, from day 0 to 7 (for 8 consecutive days) along with paclitaxel. All the animals were tested for heat and cold. The tail flick test was done around 10.00 Am, and then tail cold allodynia test was done around 12.00 PM. On day 7 intraperitoneal injection of lercanidipine was given at 8.00 AM and tail flick test was done around 10.00 AM and then tail cold allodynia test was done around 12.00 PM.

### 2.6. Assessment of Nociceptive Thresholds

#### 2.6.1. Tail Flick Method [7]

For this, analgesiometer (Techno, India) was used. The terminal (junction between proximal 2/3 and distal 1/3) portion of the tail was kept at 0.5 cm above the heated tantalums wire, and time to flick and remove the tail was noted. The tail was not kept for more than 20 seconds to avoid burn injury.

#### 2.6.2. Tail Cold Allodynia [8]

It was measured by immersing the tip of the tail in ice-cold (4°C) water and tuning the latency to a withdrawal reflex. A cut of latency of 20 seconds was kept to avoid any injury.

### 2.7. Statistical Analysis

The data was expressed as mean ± SD. Data were analyzed by using multiple comparisons ANOVA followed by Tukey's HSD test. Comparisons were made between test and control groups. A *P* value less than 0.05 was considered statistically significant.

## 3. Results

Paclitaxel-induced neuropathy was seen at 14 day. Decreased in latency was observed in tail flick and cold allodynia, and it was persisted till 28 days. No other abnormal behavioural response was seen. The effect of lercanidipine at various doses on tail flick and cold allodynia latency is presented in Tables 1 and 2. There was a significant decrease (*P* < 0.001) in the tail flick and cold allodynia latency in the paclitaxel-alone group from day 14 onward when compared with day 0. In the lercanidipine groups, the decrease in the tail flick and cold allodynia latency was not observed in 1.0 and 2.5 *μ*g/kg groups and it was statistically significant (*P* < 0.01) when compared with paclitaxel alone group. At 0.5 *μ*g/kg dose, lercanidipine was not effective in preventing neuropathy produce by paclitaxel.

## 4. Discussion

Neuropathic pain is defined as “pain initiated or caused by a primary lesion or dysfunction in the nervous system”. [1] Neuropathic pain may be divided into peripheral, central, or mixed (peripheral and central) neuropathic pain. As much as 7% to 8% of the population is affected and in 5%, it may be severe [3]. Unique to oncology is the role of chemotherapy-induced damage to peripheral sensory neurons. The precise mechanisms are unknown and likely vary with the chemotherapeutic agent administered. However, recent advances in the laboratory provide some information regarding the damage incurred to the nervous system. For example, paclitaxel administered to rats produces symptoms of peripheral neuropathy similar to those seen in humans, including hyperalgesia (an increased response to stimuli that are usually painful) and allodynia (pain due to light touch or other stimuli that are not usually painful) [9]. 

*In order to evaluate the mechanisms of neuropathic pain and to find new therapeutic approaches, different experimental neuropathic pain models have been developed which include chronic constriction injury of sciatic nerve, partial sciatic nerve ligation, and partial transaction of sciatic nerve. *Current knowledge regarding the mechanisms of neuropathic pain is incomplete and is biased by a focus on animal models of peripheral nerve injury, and the treatment is often unsatisfactory. It is an area of largely unmet therapeutic need. The current pharmacological mainstays of clinical management are tricyclic antidepressants and certain anticonvulsants, but these only achieve clinically significant (>50%) pain relief in 40–60% of patients and are associated with several side effects. Opioids are generally considered to be less effective in neuropathic pain than in inflammatory pain [4].

Paclitaxel is one of the most effective and commonly used antineoplastic drugs for the treatment of solid tumours. It has two serious side effects, myelosuppression and peripheral neurotoxicity [10]. Paclitaxel produces a dose-limiting painful peripheral neuropathy in a clinically significant number of cancer patients. Clinically, paclitaxel-induced neurotoxicity typically presents as a sensory neuropathy, with the most common complaints being numbness, tingling, and burning pain. Sensory symptoms usually start symmetrically in the feet, but sometimes appear simultaneously in both hands and feet. Most cases resolve within months after paclitaxel treatment is discontinued, but the sensory abnormalities and pain sometimes become a chronic problem. In experimental animals, paclitaxel *can produce a dose-limiting painful peripheral neuropathy when given intraperitoneally and can be used as a model of peripheral neuropathy to evaluate the effect of various drugs in neuropathic pain [5].*

*In the present study, *young Wistar rats were administered single intraperitoneal (i.p.) injection of 1 mg/Kg of paclitaxel on four alternate days (0, 2, 4, and 6) and neuropathy was seen at day 14.

Voltage-gated Ca^2+^ channels conduct Ca^2+^ ions into the neuron during depolarization and have roles in synaptic transmitter release from peripheral and central synapses, membrane excitability, and intracellular signaling events that alter gene expression and lay the foundations for LTP within spinal sensory neurons. The sensory neuron's repertoire of voltage-gated Ca^2+^ channels includes L-, N-, P/Q-, R- and T-type channels, which subserve different functional roles relative to their cellular locations. L channels are key determinants of membrane excitability, N and P/Q channels influence transmitter release at synaptic junctions, whilst R channels, which are particularly prevalent in nociceptive spinal cord pathways, confer a supporting role. T channels are low-voltage-activated channels that permit Ca^2^ flux at resting membrane potentials, hence their role in peacemaking, neuronal bursting, and synaptic-signal boosting.

Various preclinical studies point to an altered role of voltage-gated Ca^2+^ channels, particularly the *α*2*δ* subunit after neuropathy, and specific blockers have been shown to differentially attenuate the behavioral hypersensitivities and altered dorsal horn neuronal responses that accompany experimental neuropathic pain [11–13]. The studies done to evaluate the action of CCBs on CNS are quite limited. Gabapentin (GBP), a drug used in the clinical treatment of neuropathic pain, binds to the *α*2*δ* subunit of voltage-gated Ca^2+^ channels and attenuates transmitter release [14]. Lercanidipine is a third-generation L-type calcium channel blocker (CCB) belonging to the class dihydropyridines and is approved for the treatment of cardiovascular disorders. A single study conducted in the Department of Pharmacology, PGIMER, Chandigarh, reported the efficacy of lercanidipine in orofacial pain model of rat [15].

Studies have demonstrated that T-type calcium channels may play a role in chemotherapy-induced neuropathy and moreover identify ethosuximide as a new potential treatment for chemotherapy-induced pain [16–18]. There are studies in the published literatures that have demonstrated that agents like neurotropin [19], ganglioside [20, 21], acetyl-L-carnitine [22] prevent and reduce paclitaxel-induced painful neuropathy in rats.

**Figure 1:**
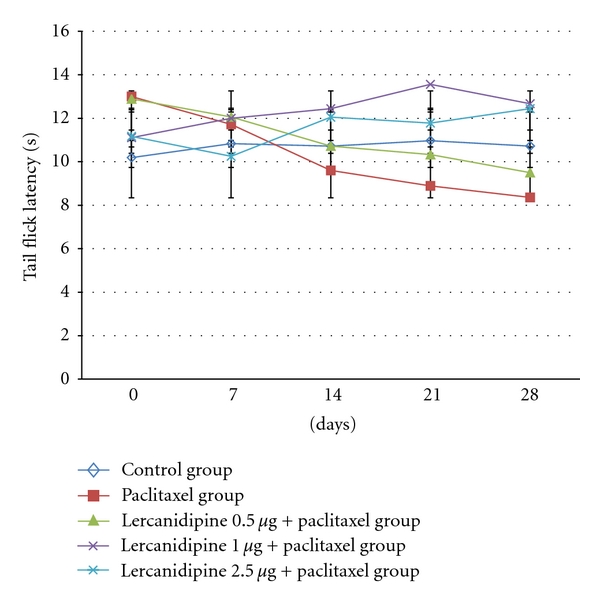
Effect of lercanidipine in various doses on cold allodynia threshold (second) in paclitaxel-induced neuropathic rats. Data are expressed as mean ± SD.

**Figure 2:**
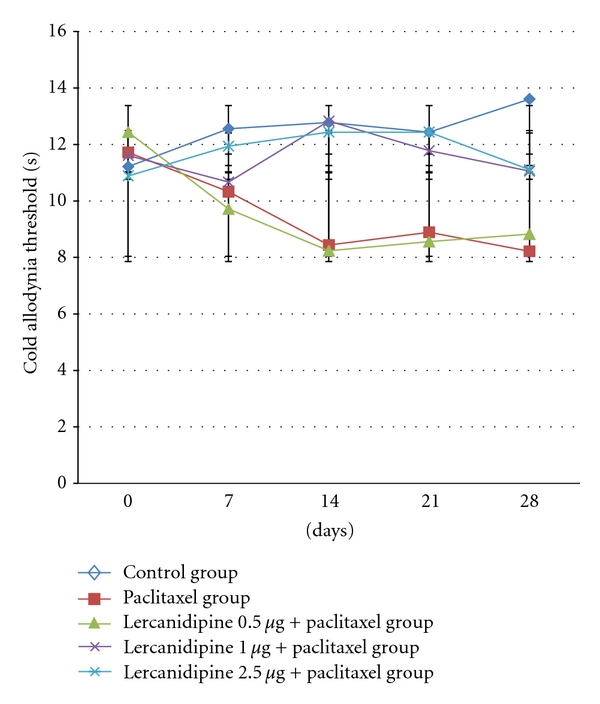
Effect of lercanidipine in various doses on cold allodynia threshold (second) in paclitaxel-induced neuropathic rats. Data are expressed as mean ± SD.

*In the present study, *paclitaxel-induced neuropathy was seen at day 14. Decrease in latency was observed in tail flick and cold allodynia, and it was persisted till 28 days. Three doses (0.5, 1.0, 2.5 *μ*g/kg) of lercanidipine were used to prevent paclitaxel-induced neuropathy in the present study, and both of the 1.0, 2.5 *μ*g/kg doses were effective in preventing neuropathy. To the best of our knowledge, the present study is the first study to demonstrate the effect of lercanidipine in paclitaxel-induced neuropathy model in rat (Figures 1 and 2). But the limitation of the present study is that we have not explored the underlying mechanism of lercanidipine. This is the pilot study with lercanidipine, and the result provide the view that lercanidipine may be an option for preventing paclitaxel-induced neuropathy. Further studies with this drug in different neuropathic models and receptor characterization should be done, which may provide a better answer.

## 5. Conclusion

*Lercanidipine *is a third-generation L-type calcium channel blocker (CCB) belonging to the class dihydropyridines and is approved for the treatment of cardiovascular disorders. To the best of our knowledge, the present study is the first pilot study to demonstrate the effect of lercanidipine in paclitaxel-induced neuropathy model in rat, and the results provide the view that lercanidipine may be an option for preventing paclitaxel-induced neuropathy. Further studies with this drug in different neuropathic models and receptor characterization should be done, which may provide a better answer.

## Figures and Tables

**Table 1:** Effect of lercanidipine in various doses on Tail flick latency (second) in paclitaxel-induced neuropathic rats. Data are expressed as mean ± SD.

Day	Control (*n* = 6)	Paclitaxel 1 mg/kg (*n* = 6)	Lercanidipine 0.5 *μ*g/kg (*n* = 6)	Lercanidipine 1.0 *μ*g/kg (*n* = 6)	Lercanidipine 2.5 *μ*g/kg (*n* = 6)
0	10.19 ± 0.73	13 ± 1.55	12.89 ± 1.63	11.11 ± 1.52	11.17 ± 1.28
7	10.83 ± 1.26	11.72 ± 1.22	12.06 ± 0.83	12.00 ± 1.19	10.25 ± 0.87
14	10.72 ± 1.27	9.6 ± 1.02*	10.72 ± 1.31*	12.44 ± 2.04^#^	12.05 ± .75^#^
21	10.97 ± 1.31	8.86 ± 0.98*	10.33 ± 1.31*	13.56 ± 1.93^#^	11.78 ± 2.05^#^
28	10.72 ± 2.04	8.36 ± .64*	9.5 ± .68*	12.67 ± 1.05^#^	12.44 ± 1.38^#^

*P < 0.001 compared to 0 day, ^#^ *P* < 0.01 compared to paclitaxel group.

**Table 2:** Effect of lercanidipine in various doses on cold allodynia threshold (second) in paclitaxel-induced neuropathic rats. Data are expressed as mean ± SD.

Day	Control (*n* = 6)	Paclitaxel 1 mg/kg (*n* = 6)	Lercanidipine 0.5 *μ*g/kg (*n* = 6)	Lercanidipine 1.0 *μ*g/kg (*n* = 6)	Lercanidipine 2.5 *μ*g/kg (*n* = 6)
0	11.22 ± 2.09	11.72 ± 1.56	12.44 ± 2.4	11.61 ± 2.33	10.89 ± 1.54
7	12.56 ± 1.2	10.33 ± 1.32	9.72 ± 1.08	10.67 ± 2.15	11.94 ± 1.40
14	12.78 ± 2.86	8.44 ± 2.14*	8.24 ± 2.70*	12.83 ± 1.38^#^	12.44 ± 1.44^#^
21	12.44 ± 2.41	8.89 ± 2.22*	8.56 ± 3.14*	11.78 ± 1.54^#^	12.44 ± 1.96^#^
28	13.61 ± .85	8.22 ± 2.28*	8.83 ± 2.87*	11.06 ± 1.85^#^	11.11 ± .54^#^

*P < 0.001 compared to 0 day, ^#^ *P* < 0.01 compared to paclitaxel group.
